# NOD2 deficiency exacerbates hypoxia-induced pulmonary hypertension and enhances pulmonary vascular smooth muscle cell proliferation

**DOI:** 10.18632/oncotarget.23912

**Published:** 2018-01-03

**Authors:** Min-Young Kwon, Narae Hwang, Young-Jun Park, Mark A. Perrella, Su Wol Chung

**Affiliations:** ^1^ School of Biological Sciences, College of Natural Sciences, University of Ulsan, Ulsan, South Korea; ^2^ Metabolic Regulation Research Center, Korea Research Institute of Bioscience and Biotechnology, Yuseong-gu, Daejeon, South Korea; ^3^ Division of Pulmonary and Critical Care, Department of Medicine, and Department of Pediatric Newborn Medicine, Brigham and Women's Hospital and Harvard Medical School, Boston, MA, USA

**Keywords:** pulmonary hypertension, pulmonary artery smooth muscle cells, NOD2, hypoxia, HIF-1α

## Abstract

Expression of nucleotide-binding oligomerization domain protein 2 (NOD2) is upregulated in pulmonary artery smooth muscle cells (PASMCs) during hypoxia. To investigate the involvement of NOD2 in the pulmonary vascular response to hypoxia, we subjected wild-type and NOD2-deficient mice to chronic normobaric hypoxic conditions. Compared to wild-type mice, NOD2-deficient mice developed severe pulmonary hypertension with exaggerated elevation of right ventricular systolic pressure, profound right ventricular hypertrophy and striking vascular remodeling after exposure to hypoxia. Pulmonary vascular remodeling in NOD2-deficient mice was characterized by increased PASMC proliferation. Furthermore, hypoxia-inducible factor-1α expression and Akt phosphorylation were upregulated in PASMCs from NOD2-deficient mice exposed to hypoxia. Our findings revealed that the absence of NOD2 exacerbated hypoxia-induced PASMC proliferation, pulmonary hypertension and vascular remodeling, but had no effect on PASMC migration or contractility.

## INTRODUCTION

Pulmonary hypertension (PH) commonly occurs in populations that live at high altitudes and in individuals who experience chronic hypoxia due to pulmonary conditions. In people with chronic obstructive pulmonary disease, PH can cause the right side of the heart to fail, resulting in fatality [1–3]. The walls of pulmonary blood vessels can undergo cellular and histological alterations due to sustained hypoxia. The remodeling of pulmonary blood vessels is characterized by increases in pulmonary vessel wall thickness and small artery muscularization. *In vivo* studies have indicated that hypoxia increases the wall thickness of pulmonary blood vessels by promoting pulmonary artery smooth muscle cell (PASMC) proliferation [4, 5]. Additionally, pulmonary vascular cells have been reported to proliferate in response to hypoxia *in vitro* [6–8].

Hypoxia activates phosphatidylinositol 3-kinase (PI3K) signal transduction, which leads to the phosphorylation of the serine/threonine kinase Akt. While the induction of PI3K and Akt signaling is important for cells to grow, proliferate, migrate and survive [9], this pathway can also stimulate cellular proliferation in response to hypoxia. Indeed, Akt activation has been observed during the remodeling of blood vessels and the proliferation of PASMCs following vascular injury and hypoxia [10], and has been reported to stabilize hypoxia-inducible factor-1 alpha (HIF-1α) [11]. Notably, HIF-1 and HIF-2 are important transcriptional regulators in response to hypoxia. The transcription factor HIF-1 is a heterodimer: α-subunit (HIF-1α) expression depends on oxygen levels, while β-subunit (HIF-1β/ARNT) expression is constitutive [12]. During normoxia, the degradation of HIF-1α is initiated by the hydroxylation of conserved proline residues [13]. However, during hypoxia, this process is inhibited, such that HIF-1α accumulates, dimerizes with HIF-1β and induces hypoxia-specific gene expression [14]. Recently, the development of pulmonary arterial hypertension was linked to HIF signaling [5, 6, 8, 15]. The hypoxia-induced proliferation of smooth muscle cells is inhibited by HIF-1α knockdown [13], suggesting that HIF-1α contributes to the remodeling of pulmonary blood vessels and the elevation of right ventricular systolic pressure (RVSP) when PASMCs are exposed to chronic hypoxia.

The nucleotide-binding oligomerization domain protein 2 (*NOD2*) gene has been reported to determine whether individuals will be vulnerable to inflammatory bowel diseases and Crohn's disease [16, 17]. This gene encodes an intracellular protein that resembles the Toll-like receptors (TLRs) in its leucine-rich repeats. Through its binding to muramyl dipeptide, NOD2 functions intracellularly as a pathogen recognition receptor (PRR) that recognizes the peptidoglycans of gram-positive and -negative bacteria [18]. NOD2 is expressed in myeloid cells such as neutrophils, macrophages and dendritic cells, and in small intestinal Paneth cells [19, 20]. However, NOD2 has been reported to be expressed or to perform different activities in cells such as vascular endothelial cells, gingival and pulp fibroblasts, adipocytes and vascular smooth muscle cells (VSMCs) [21–26].

The function of NOD2 in systemic arterial VSMCs has been published, but its function in VSMCs of the pulmonary circulatory system (including the remodeling of pulmonary blood vessels) has been underexplored. Previously, we demonstrated that arterial VSMCs expressed functional NOD2, but were stimulated to proliferate, migrate and form neointima in a NOD2-deficient animal model [26]. Here, we evaluated the effects of NOD2 on HIF-1α and Akt signaling, pulmonary vascular remodeling and PASMC proliferation in response to hypoxia.

## RESULTS

### NOD2 deficiency increases RVSP and induces right ventricular hypertrophy following chronic hypoxia

We first investigated the involvement of NOD2 in the pulmonary reaction to hypoxic conditions by analyzing the mRNA expression of *Nod2* within mouse lungs. *Nod2* expression has been reported in many different cell types, including systemic arterial VSMCs [26]. VSMCs are also very important in the pulmonary circulatory system, and perform a number of tasks that are critical for pulmonary function. However, the contribution of NOD2 to lung disease has yet to be reported. We compared the mRNA levels of *Nod2* in the lungs of wild-type mice with those in bone marrow-derived macrophages (BMDMs) stimulated with LPS and IFN-gamma to induce *Nod2* expression (a positive control) (Supplementary Figure 1). Notably, the basal level of *Nod2* expression was 12-fold higher in mouse lungs than in BMDMs.

**Figure 1:**
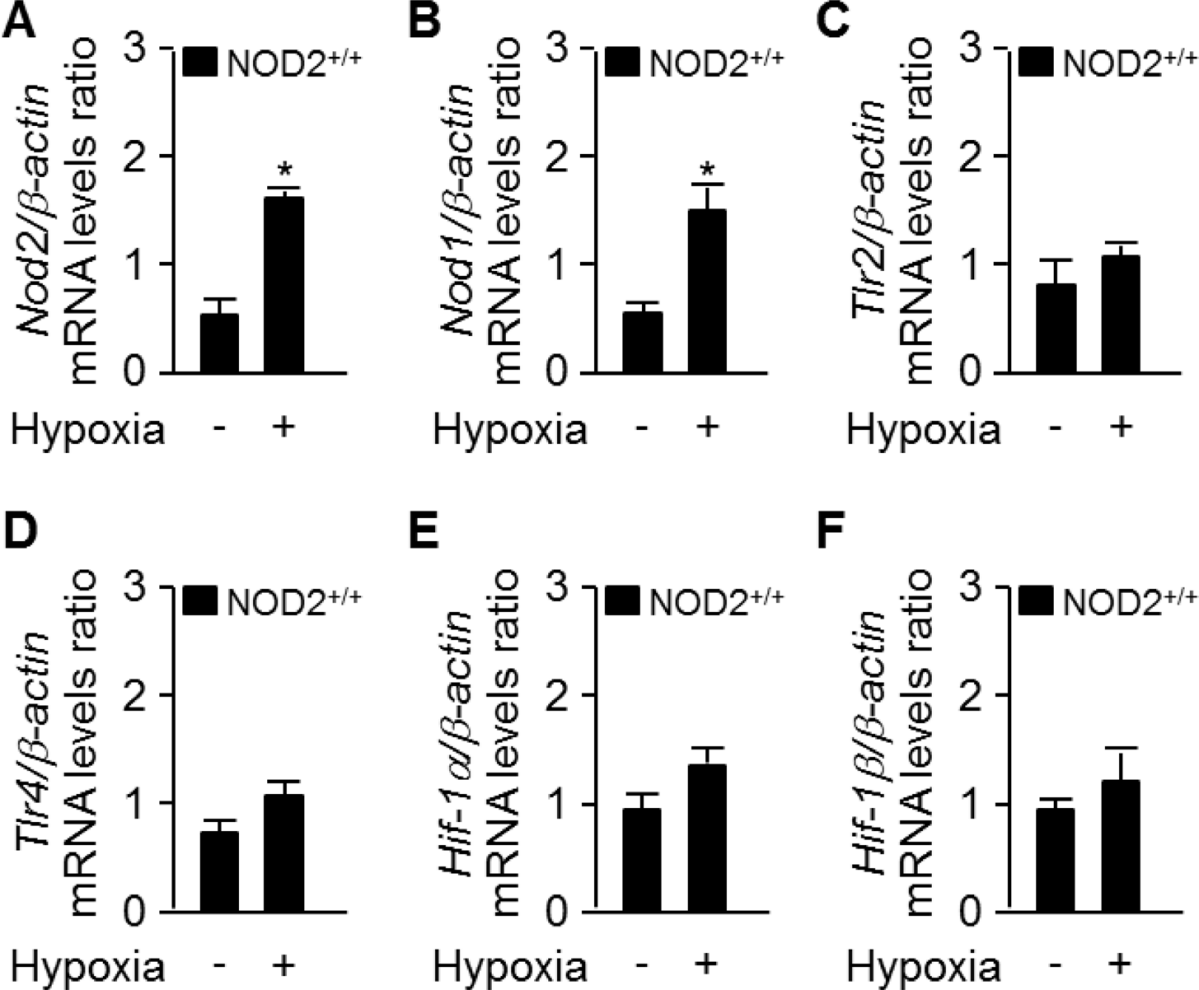
Nod2 expression is enhanced in the mouse lung after chronic hypoxia -Total RNA was extracted from NOD2^+/+^ lungs after two weeks of exposure to normoxic (21% O_2_) or normobaric hypoxic (10% O_2_) conditions. Quantitative real-time RT-PCR was performed to compare the mRNA levels of *Nod2* (**A**), *Nod1* (**B**), *Tlr2* (**C**), *Tlr4* (**D**), *Hif-1α* (**E**) and *Hif-1*β (**F**) between the groups. ^*^P < 0.05, upregulation of mRNA levels in the hypoxia vs. normoxia group. For all real-time PCR analyses, mouse β*-actin* was used as a control for normalization. The expression of each mRNA was normalized to that of β*-actin*. Values are presented as means ± SDs, *n* = 3.

Hypoxia is a well-known stimulus of PH, and can occur in individuals with extensive damage of the lung parenchyma due to chronic lung conditions [27]. Hence, we investigated whether hypoxia stimulates the expression of *Nod2*. We extracted total RNA from NOD2^+/+^ mouse lungs after exposing them to normoxic or hypoxic conditions for two weeks. As shown in Figure 1A and 1B, the mRNA levels of *Nod2* and *Nod1* in the lungs were greater in mice subjected to hypoxia than in those exposed to normoxia. However, there were no differences between the two groups in *Tlr2*, *Tlr4*, *Hif-1α* or *Hif-1β* mRNA levels after two weeks (Figure 1C–1F).

**Figure 2:**
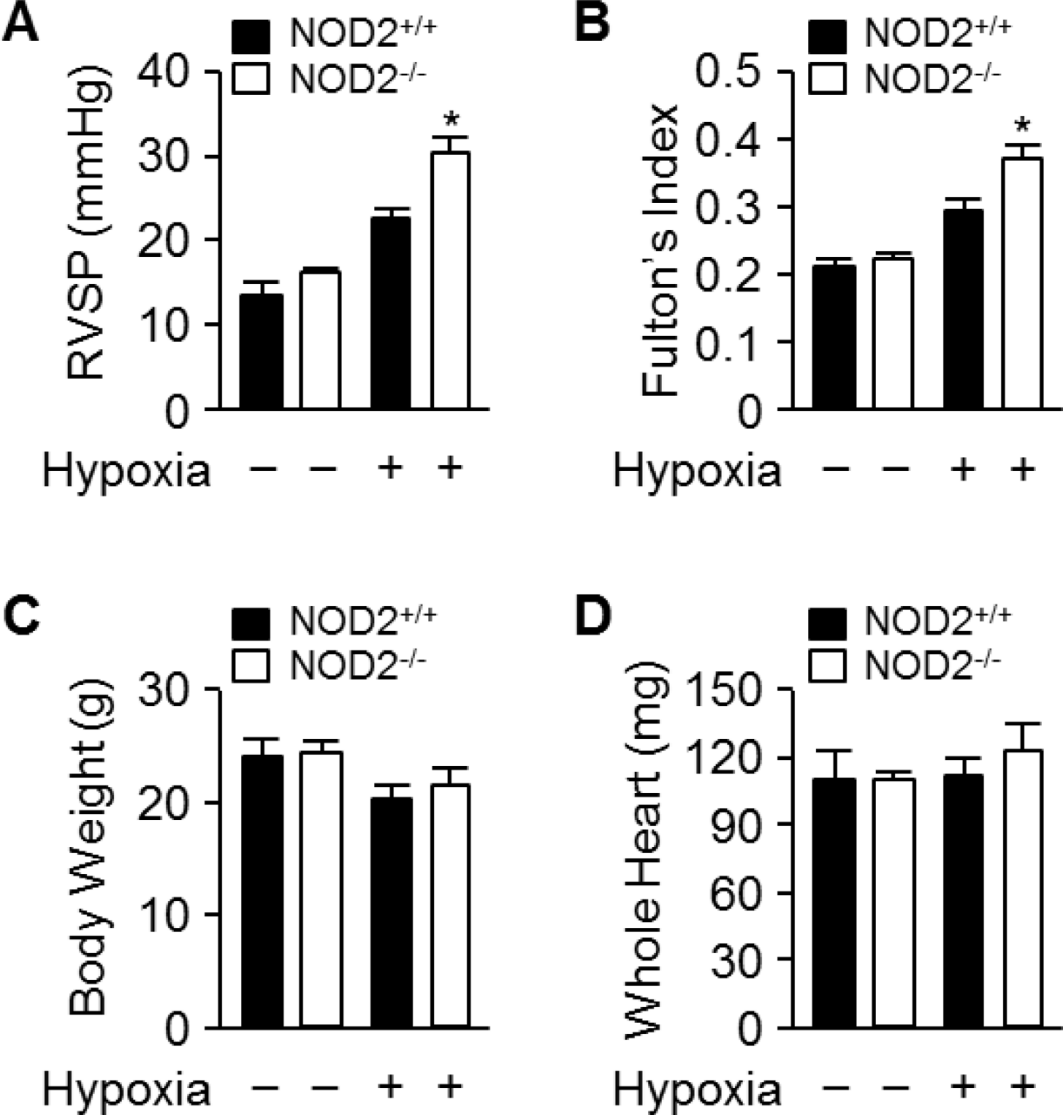
Exaggerated elevation of RVSP and Fulton’s index values in NOD2^−/−^ mice after chronic hypoxia (**A**) RVSP in NOD2^+/+^ (black bars) and NOD2^−/−^ (white bars) mice after two-week exposure to hypoxic (*n* = 18 per group) or normoxic (*n* = 10 per group) conditions. (**B**) Fulton’s index values (ratio of right ventricular weight to left ventricular plus septal weight) of NOD2^+/+^ (black bars) and NOD2^−/−^ (white bars) mice after two-week exposure to hypoxic (*n* = 18 per group) or normoxic (*n* = 15 per group) conditions. C and D, The body weights (**C**) and whole heart weights (**D**) of NOD2^+/+^ (black bars) and NOD2^−/−^ (white bars) mice after exposure to hypoxic (*n* = 18 per group) or normoxic (*n* = 15 per group) conditions for two weeks. Data are expressed as means ± SDs; ^*^P < 0.05 for hypoxic NOD2^−/−^ mice vs. hypoxic NOD2^+/+^ mice.

Next, we subjected NOD2^−/−^ and NOD2^+/+^ mice to normoxic or normobaric hypoxic conditions for two weeks. We then assessed whether the pulmonary arteries were becoming hypertensive by determining the RVSP and Fulton's index (the ratio of the right ventricular mass to the sum of the left ventricular and septal masses), a measure of hypertrophy in the right ventricle due to elevated right ventricular pressure and afterload. Following hypoxic treatment, the RVSP was significantly higher in NOD2^−/−^ mice (30.13 ± 0.9 mmHg) than in NOD2^+/+^ mice (23.6 ± 1.3 mmHg; *P* < 0.05) and normoxic mice (Figure 2A). Additionally, Fulton's index values were higher in NOD2^−/−^ mice than in NOD2^+/+^ mice (0.37 ± 0.002 vs. 0.29 ± 0.003, *P* < 0.05) after normobaric hypoxic treatment (Figure 2B). However, the total body weights and whole heart weights did not differ between NOD2^−/−^ (21.59 ± 0.4 g and 111.53 ± 3.4 mg, respectively) and NOD2^+/+^ (20.27 ± 0.75 g and 122.12 ± 5.6 mg, respectively) mice after exposure to normobaric hypoxic conditions (Figure 2C and 2D). Our results indicate that increased NOD2 levels improve the reaction of pulmonary blood vessels to hypoxic conditions.

Absence of NOD2 promotes the remodeling of pulmonary blood vessels and the hypertrophy of PASMCs following chronic hypoxia.

**Figure 3:**
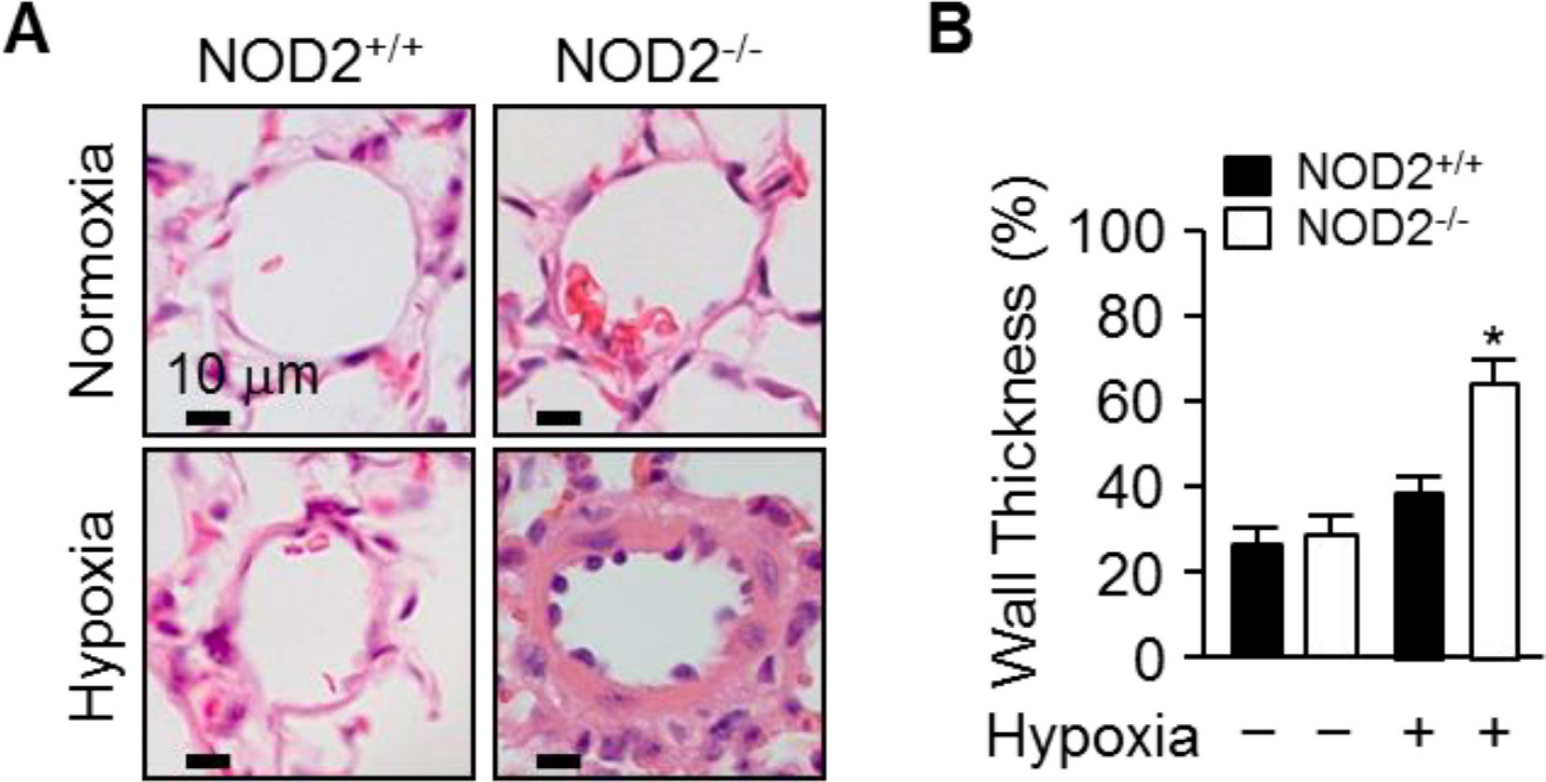
NOD2 deficiency enhances pulmonary vascular remodeling after chronic hypoxia (**A**) Representative 5-μm hematoxylin and eosin-stained sections from NOD2^+/+^ (left) and NOD2^−/−^ (right) mice, generated after two weeks of normoxic (top) or hypoxic (bottom) treatment. (**B**) Quantification of pulmonary arteriole wall thickness within the lungs of NOD2^+/+^ (black bars) and NOD2^−/−^ (white bars) mice after exposure to normoxic (*n* = 7 per group) or hypoxic (*n* = 10 per group) conditions for two weeks (values expressed as percentages). Ten vessels were analyzed per mouse. Data are expressed as means ± SDs; ^*^P < 0.05 for hypoxic NOD2^−/−^ mice vs. hypoxic NOD2^+/+^ mice.

We then performed histology to investigate the effects of NOD2 expression on the remodeling of pulmonary blood vessels during PH caused by hypoxia. Staining with hematoxylin and eosin demonstrated that the blood vessels were remodeled to a greater extent in NOD2^−/−^ mice than in NOD2^+/+^ mice after hypoxic treatment (Figure 3A). Moreover, under hypoxic conditions, the thickness of the pulmonary arteriole walls increased further in NOD2^−/−^ mice (64.19 ± 6.8%) than in NOD2^+/+^ mice (38.85 ± 3.6%; *P* < 0.05) or normoxic controls (Figure 3B).

**Figure 4:**
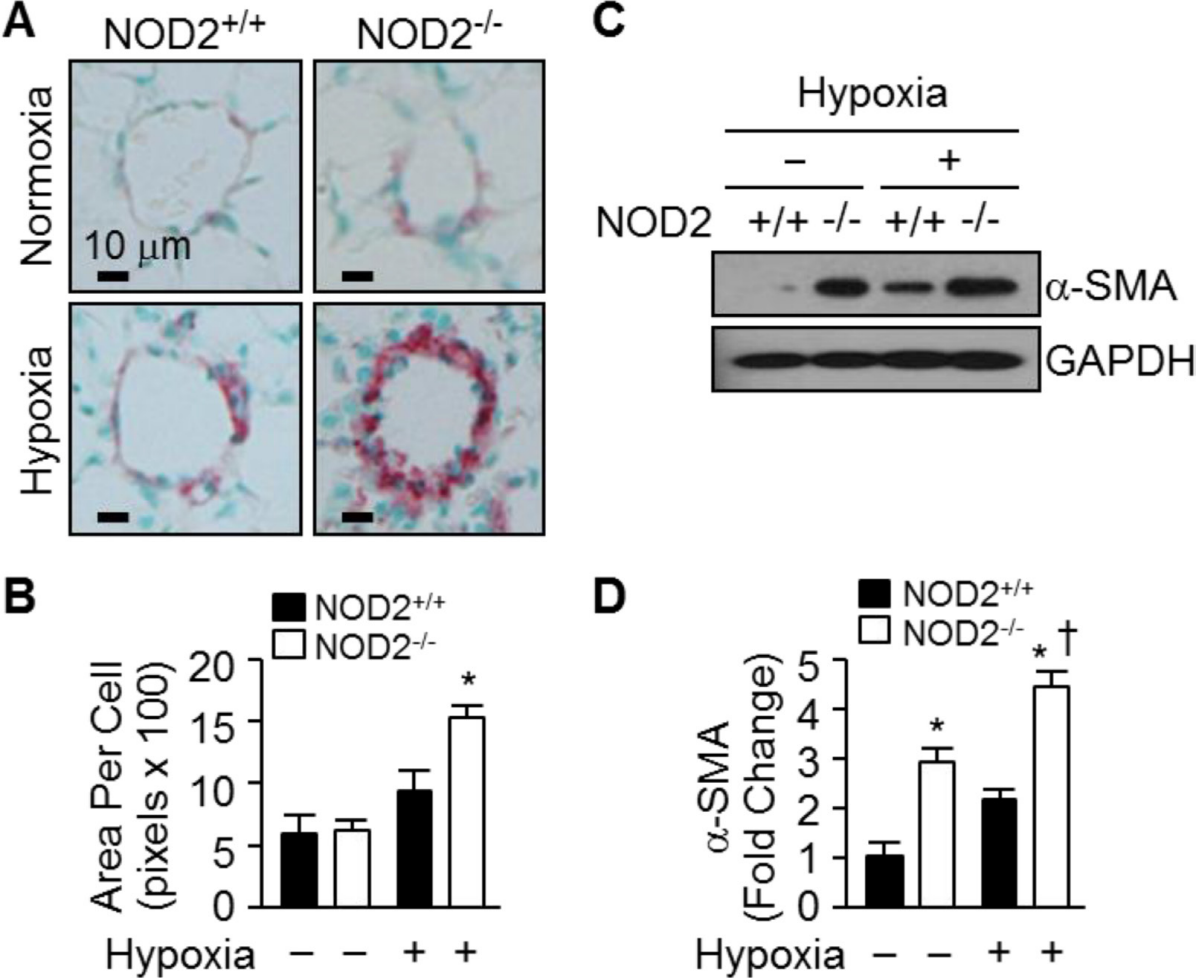
NOD2^−/−^ mice exhibit enhanced hypertrophy of PASMCs after hypoxia (**A**) α-SMA immunostaining of the lungs of NOD2^+/+^ (left) and NOD2^−/−^ (right) mice after exposure to normoxic (top) or hypoxic (bottom) conditions for two weeks. (**B**) Quantification of PASMC size in NOD2^+/+^ (black bars) and NOD2^−/−^ (white bars) mice. Ten vessels per mouse were analyzed after exposure to normoxic (*n* = 5 per group) or hypoxic (*n* = 8 per group) conditions for two weeks. Data are expressed as means ± SDs; ^*^P < 0.05 for hypoxic NOD2^−/−^ mice vs. hypoxic NOD2^+/+^ mice. (**C**) Western blot analysis of α-SMA levels in the lungs of NOD2^+/+^ and NOD2^−/−^ mice after two weeks of exposure to hypoxic or normoxic conditions. GAPDH was used as control for normalization. The experiment was conducted three times, and the images depict representative results. (**D**) Normalized α-SMA levels represented graphically. ^*^P < 0.05 for hypoxic mice vs. normoxic controls; ^†^ *P* < 0.05 for hypoxic NOD2^−/−^ mice vs. hypoxic NOD2^+/+^ mice.

To determine whether the enhanced pulmonary vascular remodeling after normobaric hypoxia involved PASMC hypertrophy, we analyzed the prevalence of alpha-smooth muscle actin (α-SMA)-positive cells in the lungs of NOD2^−/−^ and NOD2^+/+^ mice. In both NOD2^−/−^ and NOD2^+/+^ mice, little collagen was deposited in the distal blood vessels that had undergone remodeling (Trichrome staining; data not shown). On the other hand, α-SMA immunostaining indicated that the blood vessels of NOD2^−/−^ mice were profoundly remodeled in response to hypoxic conditions; the distal pulmonary arterioles were neomuscularized, exhibiting neointimal enlargement and α-SMA-positivity (Figure 4A). Conversely, the vessels of NOD2^+/+^ mice were remodeled to a significantly lesser degree and harbored fewer α-SMA-positive cells. Morphometric analysis revealed significant PASMC hypertrophy after hypoxia in NOD2^−/−^ mice, corresponding to a significantly greater area per cell (1540 ± 98 pixels) than their NOD2^+/+^ counterparts (950 ± 158 pixels) (Figure 4B).

Subsequently, we performed Western blotting to quantify the amount of α-SMA protein in the lungs of NOD2^−/−^ and NOD2^+/+^ mice after exposing them to hypoxic and normoxic conditions (Figure 4C). Interestingly, the baseline levels of α-SMA protein were slightly higher in NOD2^−/−^ mice than in NOD2^+/+^ mice. After hypoxia, however, α-SMA protein expression increased an additional 4.5-fold in NOD2^−/−^ lungs, significantly greater than the 2.2-fold upregulation observed in NOD2^+/+^ lungs (Figure 4D). Thus, NOD2 may confer protective effects with respect to pulmonary vascular remodeling and PASMC hypertrophy after hypoxia.

### Enhanced PASMC proliferation contributes to the hypoxic vascular remodeling of the NOD2^−/−^ strain

**Figure 5:**
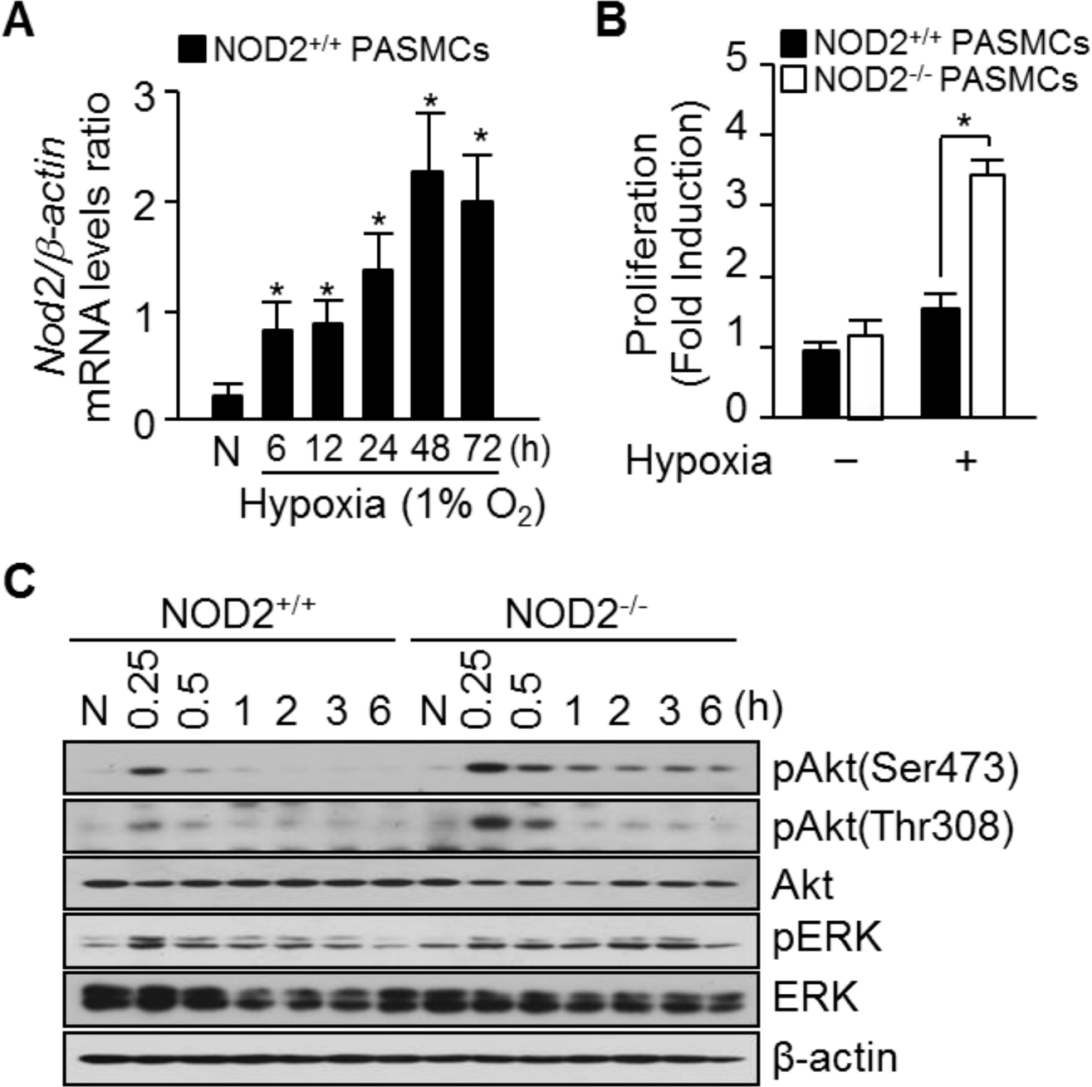
Absence of NOD2 during hypoxia enhances PASMC proliferation (**A**) Total RNA was extracted from NOD2^+/+^ PASMCs exposed to normoxic (N) or hypoxic conditions for the indicated lengths of time. *Nod2* mRNA levels were analyzed by quantitative real-time RT-PCR, and mouse β*-actin* was used as a control for normalization. Values are presented as means ± SDs, *n* = 3. (**B**) NOD2^+/+^ and NOD2^−/−^ PASMCs were plated and incubated for 72 hours under normoxic (21% O_2_) or hypoxic (1% O_2_) conditions. Proliferation was then assessed with a BrdU Cell Proliferation Assay Kit. ^*^P < 0.05, increased cell proliferation of NOD2^−/−^ vs. NOD2^+/+^ PASMCs in response to hypoxia. Values are presented as means ± SDs, *n* = 12. (**C**) Total protein was extracted from NOD2^+/+^ and NOD2^−/−^ PASMCs exposed to normoxic (N) or hypoxic conditions for the indicated lengths of time. The levels of Akt, phosphorylated Akt (Ser473 and Thr308), total ERK and pERK were then assessed by Western blot; β-actin was used as a loading control. Experiments were performed independently at least three times.

We next investigated the function of NOD2 *in vitro* by subjecting PASMCs to hypoxic conditions and analyzing *Nod2* mRNA expression. While *Nod2* mRNA levels began to increase in PASMCs 6 hours after the cells were exposed to hypoxia, this increase was much more striking after 48 hours (Figure 5A).

To elucidate the potential mechanisms whereby NOD2 influences pulmonary vascular remodeling, we investigated the proliferation, migration and contractility of NOD2^−/−^ PASMCs exposed to hypoxia *in vitro*. The proliferation of NOD2^−/−^ PASMCs was 2.23 ± 0.42-fold greater than that of NOD2^+/+^ PASMCs under hypoxic conditions (Figure 5B). We subsequently used gelatin-coated 24-Transwell chambers to assess the effect of NOD2 on cellular migration. Notably, the migration of NOD2^−/−^ and NOD2^+/+^ PASMCs did not differ significantly (132.97 ± 4.2 cells/mm^2^ and 92.64 ± 5.7 cells/mm^2^, respectively) in response to hypoxia (Supplementary Figure 2A and 2B). Next, we investigated cell contractility using a three-dimensional collagen matrix assay. Hypoxic NOD2^−/−^ and NOD2^+/+^ PASMCs exhibited similar levels of gel contraction 4 hours after matrix release (64 ± 3.0% and 56 ± 3.3% of original gel size, respectively) (Supplementary Figure 2C and 2D).

To determine the molecular pathways that induced the growth of NOD2-deficient PASMCs, we investigated the effects of NOD2 deficiency on PI3K and Akt signaling under hypoxic conditions. For these experiments, total protein was harvested from NOD2^+/+^ and NOD2^−/−^ PASMCs at 0.25, 0.5, 1, 2, 3 and 6 hours after hypoxic treatment. Assessment of the mitogen-activated protein kinase pathway revealed that phosphorylated extracellular signal-regulated kinase (pERK) levels in NOD2^−/−^ PASMCs were not altered compared with NOD2^+/+^ PASMCs after hypoxia. Also, p-p38 and stress-activated protein kinase/c-Jun NH_2_-terminal kinase (SAPK/JNK) were not detected in response to hypoxia (data not shown). In contrast, Akt phosphorylation (i.e., activation) was enhanced in NOD2^−/−^ PASMCs exposed to hypoxic conditions compare with NOD2^+/+^ PASMCs (Figure 5C).

**Figure 6:**
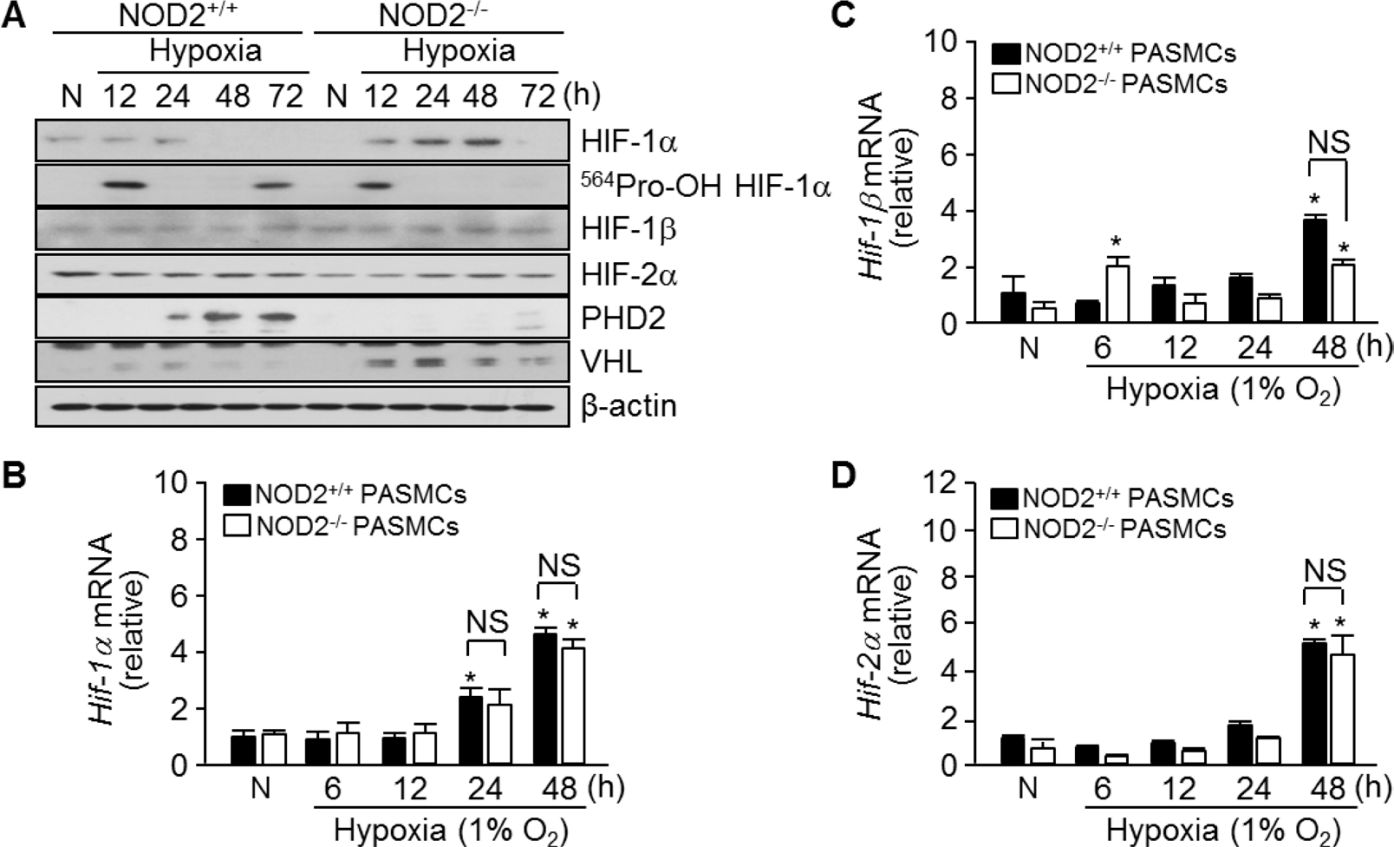
Absence of NOD2 enhances the stability of HIF-1α protein in PASMCs exposed to hypoxic conditions (**A**) Total protein was extracted from NOD2^+/+^ and NOD2^−/−^ PASMCs exposed to normoxic (N) or hypoxic conditions for the indicated lengths of time. The protein levels of HIF-1α, hydroxylated HIF-1α (Pro564), HIF-1β, HIF-2α, PHD2 and VHL were then assessed by Western blot; β-actin was used as a loading control. Experiments were performed at least three independent times. (**B**–**D**), Total RNA was extracted from NOD2^+/+^ and NOD2^−/−^ PASMCs exposed to normoxic (N) or hypoxic conditions for the indicated lengths of time. The mRNA levels of *Hif-1α* (B), *Hif-1*β (C) and *Hif-2α* (D) were then analyzed by quantitative real-time RT-PCR; mouse β*-actin* was used as a control for normalization. ^*^P < 0.05, upregulation after hypoxia vs. after normoxia (N). NS, not significant. Values are presented as means ± SDs, *n* = 3.

HIF-1α is another important mediator of arterial remodeling and PH in response to chronic hypoxia [15]. To investigate the effects of NOD2 deficiency on HIF-1α expression, we harvested total RNA and protein from NOD2^+/+^ and NOD2^−/−^ PASMCs exposed to hypoxia for 6, 12, 24, 48 and 72 hours. HIF-1α protein was maintained until 24 hours post-exposure and then it was decreased in NOD2^+/+^ PASMCs. On the other hand, an increase in the expression of this protein was observed in NOD2^−/−^ PASMCs by 12 hours after hypoxia exposure, and a more striking increase was evident after 48 hours (Figure 6A). Furthermore, in NOD2^−/−^ PASMCs, we observed reduced protein expression of hydroxyl-HIF-1α (Pro564) and prolyl hydroxylase domain protein 2 (PHD2), which facilitates the attachment of HIF-1α to the von Hippel-Lindau (VHL) E3 ubiquitin ligase and its subsequent ubiquitin-dependent degradation [13, 28]. Overall, however, there were no changes in HIF-1β and HIF-2α protein expression. Interestingly, increased VHL protein expression was detected in NOD2^−/−^ PASMCs. Lastly, we observed a time-dependent increase in *Hif-1α*, *Hif-1β* and *Hif-2α* mRNA levels in both NOD2^+/+^ and NOD2^−/−^ PASMCs upon exposure to hypoxic conditions; however, the expression of these factors did not differ between NOD2^+/+^ and NOD2^−/−^ PASMCs after hypoxia exposure (Figure 6B–6D).

Together, our data indicate that greater PASMC proliferation, not migration or contractility, is responsible for the remodeling of pulmonary blood vessels in hypoxia-exposed NOD2^−/−^ mice. Moreover, prolonged Akt phosphorylation and HIF-1α expression are important contributors to the enhanced proliferation of NOD2^−/−^ PASMCs during hypoxia.

## DISCUSSION

TLRs, which comprise a class of transmembrane PRR, were suggested to connect inflammation with PH, thus contributing to the emergence of PH, while being dispensable for lesion formation [29–31]. TLR signaling, especially involving TLR2 and 4, has been described in human pulmonary vascular cells and patients with idiopathic pulmonary arterial hypertension [29–32]. Moreover, the TLR Pro631His variant correlates robustly with PH incidence in patients with systemic sclerosis [30]. Conversely, other studies have suggested that NOD2 and TLR3 have protective functions in vascular diseases, including atherosclerosis [26, 33]; indeed, we previously demonstrated such a function of NOD2 in arterial VSMCs. Mouse arterial VSMC proliferation and migration were associated negatively with the induction of NOD2 expression, and NOD2-deficient arterial VSMCs proliferated, migrated and formed neointima following vascular damage [26]. Using a perivascular collar model, Cole *et al.* demonstrated that activated TLR3 suppressed the development of the neointima following carotid damage. In contrast, atherosclerotic lesions in the aortic root formed earlier in TLR3/apolipoprotein E double-knockout mice (ApoE^−/−^/TLR3^−/−^) than in control mice (ApoE^+/+^/TLR3^+/+^). Considered along with the models of vascular damage, these results indicate that PRRs like NOD2 and TLR3 may not merely be innate immune receptors, but also have other functions. While PRRs are necessary for innate immunity, they may perform markedly different activities in response to noninfectious stimuli. As an example, PRRs such as TLR3 and NOD2 protect the walls of the blood vessels, while others such as TLR2 and TLR4 can damage them. We hypothesized that NOD2 may also have a protective function during hypoxia, preventing an exaggerated increase in pulmonary vascular remodeling and RVSP.

Consistent with this hypothesis, we observed enhanced *Nod2* expression in the lungs following prolonged exposure of mice to hypoxic conditions (Figure 1A). Interestingly, the RVSP and Fulton's index values were significantly greater in NOD2-deficient mice than in wild-type mice after chronic hypoxic treatment (Figure 2A and 2B). We subsequently assessed pulmonary arterial remodeling after two weeks of normobaric hypoxic (10% O_2_) treatment to determine whether the observed changes in the RVSP and Fulton's index values were associated with PH. Following two weeks of chronic hypoxia, the medial layer of small pulmonary arteries exhibited greater thickening in NOD2^−/−^ mice than in NOD2^+/+^ mice (Figure 3A). In addition, the PASMCs of NOD2-deficient mice became significantly hypertrophic, contributing to the striking remodeling of the blood vessels (Figure 4A and 4B). Interestingly, while baseline expression of α-SMA was greater in NOD2^−/−^ mice than in NOD2^+/+^ mice, the RVSP and Fulton's index values did not differ significantly between ten- to twelve-week-old NOD2^+/+^ and NOD2^−/−^ mice exposed to normoxic conditions. Thus, in the absence of vascular remodeling (Figure 3), the source of α-SMA does not appear to be the VSMCs of small pulmonary vessels. In older mice, however, enhanced α-SMA expression might alter the RVSP or Fulton's index of NOD2^−/−^ mice under normoxic conditions.

In animals, hypoxia stimulates the proliferation of PASMCs, thus increasing the wall thickness of the pulmonary blood vessels [4, 5]. The pathologic process whereby pulmonary blood vessels are remodeled intensifies as PASMCs proliferate increasingly. Here, we used mouse primary PASMCs to study the involvement of NOD2 in PASMC proliferation in response to hypoxia. In general, hypoxia promoted *Nod2* expression in PASMCs; however, NOD2^−/−^ PASMCs proliferated at a higher level than NOD2^+/+^ PASMCs under hypoxic conditions (Figure 5A and 5B). While PASMC migration and contractility are also involved in the pathogenesis of pulmonary vascular remodeling [1, 34], the migratory and contractility capacities of NOD2^−/−^ and NOD2^+/+^ PASMCs did not differ significantly after hypoxic treatment (Supplementary Figure 2). These results suggest that NOD2 protects against pulmonary vascular remodeling under hypoxic conditions by preventing PASMC proliferation.

Generally, hypoxia initiates PI3K signaling, which leads to Akt phosphorylation [9, 10]. These pathways, along with the HIF signaling pathway, are thought to contribute to the proliferation of PASMCs under hypoxic conditions. Muramyl dipeptide, a ligand recognized by NOD2, independently induces Akt phosphorylation in different cell types [35, 36], but also inhibits insulin-stimulated Akt phosphorylation in myotubes [37]. Moreover, we previously observed sustained phosphorylation of Akt (Ser473 and Thr308) in the presence of platelet-derived growth factor-BB in NOD2^−/−^ VSMCs [26]. In the present study, we examined the influence of NOD2 on PI3K and Akt signaling to determine whether these pathways were responsible for NOD2^−/−^ PASMC proliferation under hypoxic conditions. Akt phosphorylation increased in NOD2^−/−^ PASMCs after hypoxia exposure, and a similar response was observed in the ERK signaling pathway (Figure 5C). Moreover, HIF-1α protein expression, which correlates with sustained Akt phosphorylation, was significantly higher in NOD2^−/−^ PASMCs than in NOD2^+/+^ PASMCs under hypoxic conditions (Figure 6A), although there were no differences in the mRNA levels of *Hif*-family genes between NOD2^−/−^ and NOD2^+/+^ PASMCs after hypoxic treatment (Figure 6B–6D). HIF-1α is a key protein in the lung response to reduced oxygen availability, and is thought to be involved in PH development [5, 8, 15]. Indeed, HIF-1 is necessary for smooth muscle hypertrophy, and HIF-1α significantly contributes to the remodeling of pulmonary blood vessels under chronic hypoxic conditions [8]. The hydroxylation of HIF-1α (at proline 564) and the expression of PHD2 protein were lower in NOD2^−/−^ PASMCs than in NOD2^+/+^ PASMCs after hypoxia exposure (Figure 6A). Interestingly, the levels of VHL, which participates in the ubiquitin-dependent degradation of HIF-1α, were greater in NOD2^−/−^ PASMCs than in NOD2^+/+^ PASMCs after hypoxia exposure (Figure 6A).

In conclusion, our findings indicate that NOD2 is important for the response of PASMCs to hypoxia. While we found that Akt activation leads to HIF-1α stabilization and PASMC proliferation in NOD2^−/−^ mice, future investigations will be required to characterize the molecular pathways governing the relationship between NOD2 and the Akt signaling pathway. The findings presented here have expanded our comprehension of the involvement of PRRs in hypoxia-induced PH, and demonstrate that NOD2 may be a promising target for therapies to prevent the remodeling of blood vessels in pulmonary vascular conditions.

## MATERIALS AND METHODS

### Animals

NOD2^−/−^ mice were purchased from The Jackson Laboratory (Bar Harbor, ME, USA) on a pure C57BL/6 genetic background, and these mice were bred within our animal facility at Harvard Medical School. The Standing Committee on Animal Care at Harvard Medical School approved all the animal experimentation protocols of this study under the guidelines of our approved Institutional Animal Care and Use Committee protocol.

### Hypoxic exposure and hemodynamic measurements

Eight- to ten-week-old NOD2^−/−^ and NOD2^+/+^ littermates were exposed to normobaric hypoxia (10% O_2_, OxyCycler chamber, Biospherix Ltd, Redfield, NY, USA) or normoxia (21% O_2_) for two weeks. After exposure, mice were anesthetized with sodium pentobarbital (60 mg/kg), and hemodynamic measurements were performed. The hearts were excised, and the ventricles were dissected and weighed. Right ventricular hypertrophy was calculated as the right ventricular weight normalized to the total body weight.

### Histological analysis and morphometry

Lungs were inflated, harvested, fixed in methyl Carnoy's solution and embedded in paraffin. Sections were stained with hematoxylin and eosin and immunostained for α-SMA (1:50, Sigma-Aldrich Co. LLC, St Louis, MO, USA). Remodeling was quantified as described previously [38]. The percent wall thickness was calculated as follows: wall thickness (%) = (area_ext_ − area_int_) ÷ area_ext_ × 100, where area_ext_ represents the external diameter and area_int_ represents the internal diameter of each vessel. PASMC hypertrophy was calculated as the vessel wall area divided by the number of nuclei per vessel, and reported as the area per cell.

### Cell culture

Primary PASMCs were isolated from adult (eight- to ten-week-old) NOD2^−/−^ and NOD2^+/+^ mice as described [39], with modification. Hypoxia experiments were performed in a New Brunswick™ Galaxy^®^ 48 R (1% O_2_, Eppendorf AG, Hamburg, Germany). The NOD2^−/−^ and NOD2^+/+^ PASMCs were grown in Dulbecco's Modified Eagle's Medium (Life Technologies, Grand Island, NY, USA) supplemented with 20% fetal bovine serum, penicillin (100 U/mL) and streptomycin (100 μg/mL). In all experiments, cells between passages three and nine were used. All cells were incubated at 37°C in a humidified atmosphere of 5% CO_2_ and 95% air.

### Western blot analysis

Protein extracts from lungs exposed to hypoxia or normoxia were analyzed by Western blot analysis. Cell extracts from 100-mm dishes were harvested with radioimmunoprecipitation assay buffer (Tris/Cl, pH 7.6; 100 mmol/L ethylenediaminetetraacetic acid; 5 mmol/L NaCl; 50 mmol/L β-glycerophosphate; 50 mmol/L NaF; 50 mmol/L Na_3_VO_4_; 0.1 mmol/L NP-40; and 0.5% sodium deoxycholate) with 1× Complete™ Protease Inhibitor Cocktail (Roche Applied Science, Mannheim, Germany). Tissue extracts were homogenized with radioimmunoprecipitation assay buffer. The protein concentrations of the cell lysates were determined with a Pierce BCA protein assay kit (Thermo Fisher Scientific, Inc., Waltham, MA) and were resolved on 12% sodium dodecyl sulfate-polyacrylamide gels. Proteins were transferred onto pure polyvinylidene difluoride membranes by means of a wet transfer system (GE Healthcare Bio-Sciences, Pittsburgh, PA, USA). Membranes were blocked for 2 hours at room temperature with a 5% nonfat milk solution in Tris-buffered saline Tween (TBST) buffer (20 mM Tris-HCl, pH 7.4; 500 mM NaCl; 0.1% Tween20). The blots were then incubated with various antibodies, including monoclonal α-SMA (Sigma-Aldrich Co. LLC), polyclonal anti-HIF-1α, anti-HIF-2α, anti-HIF-1β (Bethyl Laboratories, Montgomery, TX, USA), anti-phospho-Akt (Ser473, Thr308), total Akt (Cell Signaling Technology, Inc., Danvers, MA, USA), anti-PHD2, anti-VHL (Santa Cruz Biotechnology, Inc., Dallas, TX, USA) and anti-hydroxy-HIF-1α (Cell Signaling Technology), in TBST overnight at room temperature. Equal loading was confirmed with an anti-GAPDH (glyceraldehyde 3-phosphate dehydrogenase) antibody (Santa Cruz Biotechnology, Inc.) and anti-β-actin (Sigma-Aldrich Co. LLC). The blots were then washed three times in TBST and incubated with an anti-rabbit or anti-mouse secondary antibody in TBST for 1 hour at room temperature. Finally, immunoblots were detected with the SuperSignal® West Pico Chemiluminescent Substrate (Thermo Fisher Scientific, Inc.) and visualized after exposure to X-ray film.

### Proliferation assay

The cell proliferation rate at 72 hours was measured by BrdU incorporation with the Cell Proliferation ELISA, BrdU (colorimetric) kit (Roche Diagnostics, Basel, Switzerland) according to the manufacturer's instructions.

### Quantitative real-time reverse-transcription polymerase chain reaction (RT-PCR)

Total RNA was isolated with TRIzol reagent (Thermo Fisher Scientific, Inc.), and reverse transcription was performed with a SuperScript™ III First-Strand Synthesis System (Thermo Fisher Scientific, Inc.). Quantitative real-time RT-PCR was conducted with iQ SYBR Green Supermix (Bio-Rad Laboratories, Inc., Hercules, CA, USA). The primer sequences were as follows: mouse *Nod1* forward primer 5′- GAAGGCACCCCATTGGGTT -3′ and reverse primer 5′-

AATCTCTGCATCTTCGGCTGA -3′; *Nod2* forward primer 5′-

CAGGTCTCCGAGAGGGTACTG -3′ and reverse primer 5′-

GCTACGGATGAGCCAAATGAAG -3′; *Tlr2* forward primer 5′-

GCAAACGCTGTTCTGCTCAG -3′ and reverse primer 5′-

AGGCGTCTCCCTCTATTGTATT -3′; *Tlr4* forward primer 5′-

ATGGCATGGCTTACACCACC -3′ and reverse primer 5′-

GAGGCCAATTTTGTCTCCACA -3′; *Hif-1α* forward primer 5′-

TCATCAGTTGCCACTTCCCCAC -3′ and reverse primer 5′-

CCGTCATCTGTTAGCACCATCAC -3′; *Hif-1*β forward primer 5′-

TAGACCATCGTTGTGTGGCT -3′ and reverse primer 5′-

CACCTGCTGAAAGCTGTCTC -3′; *Hif-2α* forward primer 5′-

TAAAGCGGCAGCTGGAGTAT -3′ and reverse primer 5′-

ACTGGGAGGCATAGCACTGT -3′.

Amplification of cDNA started with 10 minutes at 95°C, followed by 40 cycles of 15 seconds at 95°C and 1 minute at 60°C.

### Statistical analysis

Data are shown as the mean ± standard deviation (SD). For comparisons between two groups, we used Student's two-tailed unpaired *t* test. For comparisons between more than two groups, and for multiple comparisons, we used analysis of variance. Statistically significant differences were accepted at *P* < 0.05.

## SUPPLEMENTARY MATERIALS FIGURES



## Abbreviations

α-SMA: alpha-smooth muscle actin
ApoE: apolipoprotein E
BMDMs: bone marrow-derived macrophages
ERK: extracellular signal-regulated kinase
HIF: hypoxia-inducible factor
NOD2: nucleotide-binding oligomerization domain protein 2
PASMCs: pulmonary artery smooth muscle cells
PH: pulmonary hypertension
PHD2: prolyl hydroxylase domain protein 2
PI3K: phosphatidylinositol 3-kinase
PRR: pathogen recognition receptor
RVSP: right ventricular systolic pressure
SAPK/JNK: stress-activated protein kinase/c-Jun NH_2_-terminal kinase
TLRs: toll-like receptors
VHL: von Hippel-Lindau
VSMCs: vascular smooth muscle cells.
