# Supplementary material for: NOD2 deficiency exacerbates hypoxia-induced pulmonary hypertension and enhances pulmonary vascular smooth muscle cell proliferation
Source: Oncotarget. 2018 Jan 3;9(16):12671–81. doi: 10.18632/oncotarget.23912 (PMC5849164; doi:10.18632/oncotarget.23912)
Supplement: Supplementary file 1 [file oncotarget-09-12671-s001.pdf]

NOD2 deficiency exacerbates hypoxia-induced pulmonary hypertension and enhances pulmonary vascular smooth muscle cell proliferation

SUPPLEMENTARY MATERIALS

MATERIAL AND METHODS

Bone marrow derived macrophages (BMDMs) isolation and culture

Bone marrow was flushed from femurs and tibias of C57BL/6 mice using 25-gauge needles with 10% heat-inactivated fetal bovine serum (FBS) containing PBS. Red blood cells were lysed in RBC lysis buffer (0.15 mole/L NH4Cl, 10 mmole/L KHCO3, and 0.1 mmole/L EDTA, pH 7.4). Isolated cells were incubated in RPMI 1640 Medium (Life Technologies, Grand Island, NY, USA) supplemented with 10% heat-inactivated FBS (Life Technologies, Grand Island, NY), penicillin (100 u/mL), and streptomycin (100 μg/mL), 1% glutamine, and recombinant granulocyte macrophage-colony stimulating factor (GM-CSF, 40 ng/mL). Cells were incubated at 37°C in a humidified atmosphere of 5% CO_2 and 95% air. After 5–6 days, adherent cells were washed, counted, and replated in RPMI (without GM-CSF) at a density of 5×10^6 cells/well (6-well plate, Corning, Inc., Corning, NY). To induce NOD2 expression, cells were treated with LPS (100 ng/mL, Sigma-Aldrich Co. LLC., St Louis, MO), IFN-γ (10 ng/mL, PeproTech, London, UK) for 12 hours.

Cell migration assay

Cell migration was assessed using gelatin-coated 24-well transwell chambers (Corning, Inc., Corning, NY). To assess migration, the cells were placed in the upper chamber of transwell plates and the bottom chambers were filled with 10% FBS medium. The cells were exposed to hypoxia (1% O_2). Transwell membranes were scraped and washed out to remove non-migrated cells. The cells that migrated to the lower sides of the filter membranes were fixed and stained with Crystal Violet kit (Sigma-Aldrich Co. LLC., St Louis, MO). Experiments were performed at least 3 times in duplicate.

Collagen matrix contraction assay

$NOD2^{-/-}$ and $NOD2^{+/+}$ PASMCs were plated on type I collagen gel matrices and exposed to hypoxia or normoxia for 24 hours. Gel size was defined as the sum of the 2 longest gel diameters; gel contraction was expressed as a percentage of the original gel size.

Statistical analysis

Data represent mean ± SDs. For comparisons between two groups, we used Student's two-tailed unpaired t test. For comparison between more than two groups, and multiple comparisons, we used an ANOVA test. Statistically significant differences were accepted at $p < +0.05$.

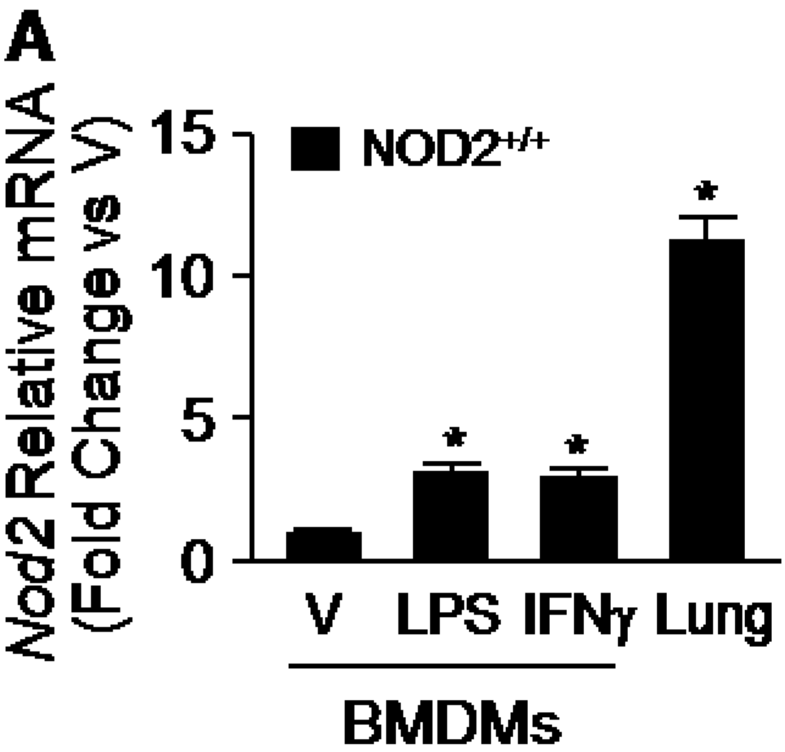

Supplementary Figure 1: Nod2 mRNA is expressed in the lung. Total RNA was extracted from lungs of $NOD2^{+/+}$ mice under normoxic conditions and from bone marrow derived macrophages (BMDMs) 12 hours after vehicle (V), LPS (100 ng/mL), or IFN-γ (10 ng/mL) administration, as a positive control. Quantitative real-time RT-PCR was performed to assess mRNA levels of Nod2 in $NOD2^{+/+}$ lung and BMDMs. $^{*}p < 0.05$, upregulation of Nod2 mRNA levels vs. vehicle-treated BMDMs. Data are expressed as mean ± SDs.

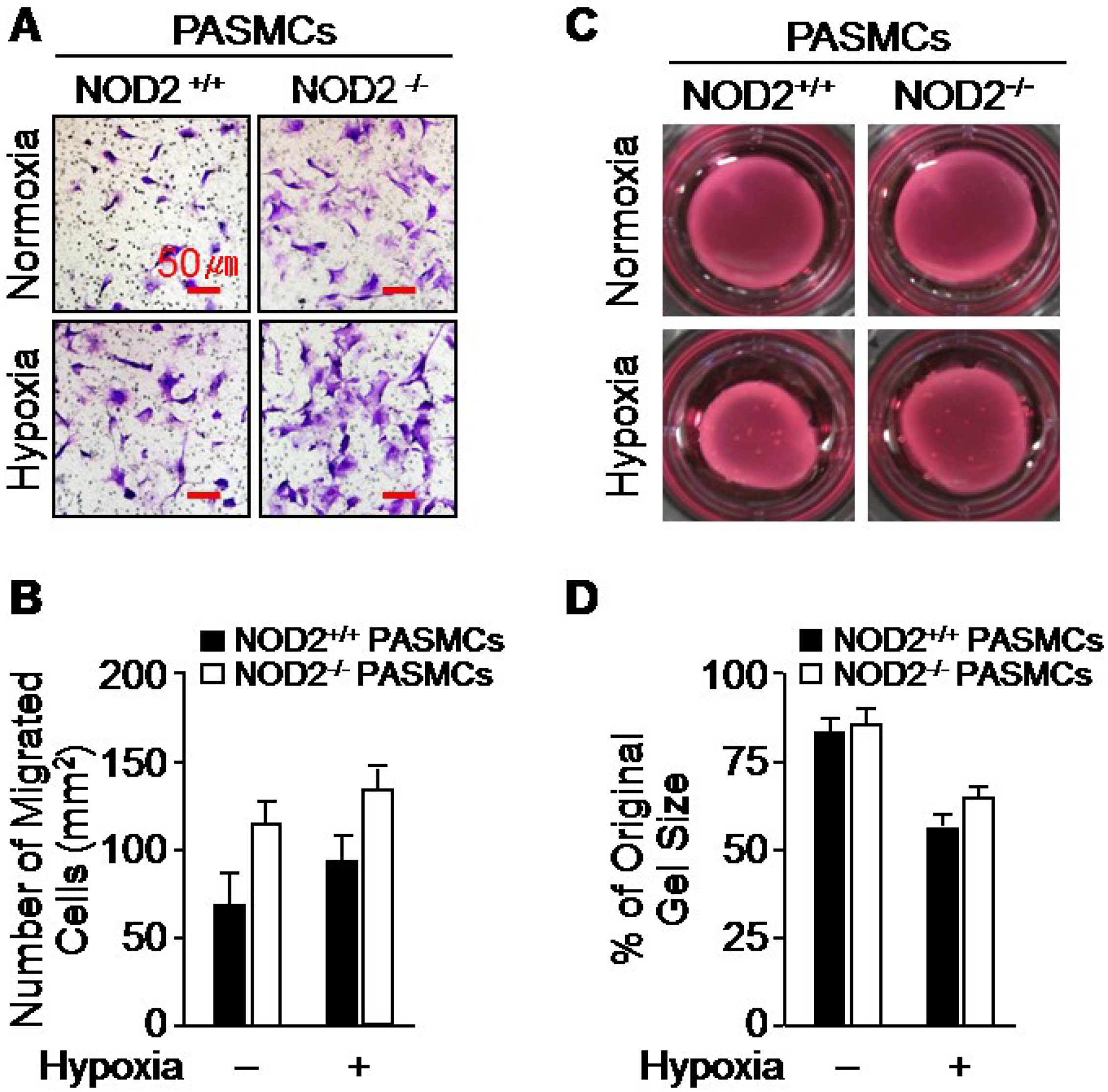

Supplementary Figure 2: Absence of NOD2 has no effects on the cell migration and contraction in PASMCs after hypoxia. (**A**) The migration assay for PASMC was analyzed using 24-Transwell chamber plates in response to normoxic (21% O2) or hypoxic (1% O2) condition. Migrated PASMCs were stained using the Crystal Violet staining kit. (**B**) The number of migrated cells was measured and is represented as a graph. Values are mean ± SDs, $n = 6$. (**C**) Representative photographs of collagen gels from $NOD2^{+/+}$ (left) and $NOD2^{-/-}$ (right) PASMCs under normoxic (top) and hypoxic (bottom) conditions. (**D**) Gel contraction after matrix release was analyzed. Data are presented as the percentage of the original collagen gel size for PASMCs ($NOD2^{+/+}$, black bars; $NOD2^{-/-}$, white bars) exposed to normoxia or hypoxia. Data are expressed as mean ± SDs.
